# Quality-Related Properties of Equine Immunoglobulins Purified by Different Approaches

**DOI:** 10.3390/toxins12120798

**Published:** 2020-12-14

**Authors:** Sanja Mateljak Lukačević, Tihana Kurtović, Maja Lang Balija, Marija Brgles, Stephanie Steinberger, Martina Marchetti-Deschmann, Beata Halassy

**Affiliations:** 1 University of Zagreb, Centre for Research and Knowledge Transfer in Biotechnology, Rockefellerova 10, HR-10000 Zagreb, Croatia; tkurtovi@unizg.hr (T.K.); maja.langbalija@gmail.com (M.L.B.); mbrgles@gmail.com (M.B.); 2 Centre of Excellence for Virus Immunology and Vaccines, CERVirVac, Rockefellerova 10, HR-10000 Zagreb, Croatia; 3 Vienna University of Technology, Institute for Chemical Technologies and Analytics, Getreidemarkt 9/164, A-1060 Vienna, Austria; stephanie.steinberger@tuwien.ac.at (S.S.); martina.marchetti-deschmann@tuwien.ac.at (M.M.-D.)

**Keywords:** plasma processing, IgG antivenom, IgG subclasses, aggregates, thermal stability

## Abstract

Whole IgG antivenoms are prepared from hyperimmune animal plasma by various refinement strategies. The ones most commonly used at industrial scale are precipitation by sodium or ammonium sulphate (ASP), and caprylic acid precipitation (CAP) of non-immunoglobulin proteins. The additional procedures, which have so far been used for experimental purposes only, are anion-exchange (AEX) and cation-exchange chromatography (CEX), as well as affinity chromatography (AC) using IgG’s Fc-binding ligands. These protocols extract the whole IgG fraction from plasma, which contains both venom-specific and therapeutically irrelevant antibodies. Such preparations represent a complex mixture of various IgG subclasses whose functional and/or structural properties, as well as relative distribution, might be affected differently, depending on employed purification procedure. The aim of this work was to compare the influence of aforementioned refinement strategies on the IgG subclass distribution, venom-specific protective efficacy, thermal stability, aggregate formation and retained impurity profile of the final products. A unique sample of *Vipera ammodytes ammodytes* specific hyperimmune horse plasma was used as a starting material, enabling direct comparison of five purification approaches. The highest purity was achieved by CAP and AC (above 90% in a single step), while the lowest aggregate content was present in samples from AEX processing. Albumin was the main contaminant in IgG preparations obtained by ASP and CEX, while transferrin dominantly contaminated IgG sample from AEX processing. Alpha-1B-glycoprotein was present in CAP IgG fraction, as well as in those from ASP- and AEX-based procedures. AC approach induced the highest loss of IgG(T) subclass. CEX and AEX showed the same tendency, while CAP and ASP had almost no impact on subclass distribution. The shift in IgG subclass composition influenced the specific protective efficacy of the respective final preparation as measured in vivo. AC and CEX remarkably affected drug’s venom-neutralization activity, in contrary to the CAP procedure, that preserved protective efficacy of the IgG fraction. Presented data might improve the process of designing and establishing novel downstream processing strategies and give guidance for optimization of the current ones by providing information on potency-protecting and purity-increasing properties of each purification principle.

## 1. Introduction

Snakebite envenoming is a medical emergency in most parts of the world, particularly serious in tropical countries where it causes significant socioeconomic problems, not only for the victims but for their entire families and communities as well [1,2]. Passive immunotherapy with animal-derived antivenoms, containing either immunoglobulin G (IgG) or its F(ab’)_2_ or Fab fragments, is already considered the only specific and effective treatment tool [2,3,4,5,6,7]. Since the progress in fighting snakebite-associated health issues represents an urgent necessity, improvements in availability, safety, and efficacy of antivenoms lately constitute one of the main WHO strategic focus areas [8]. The highly challenging task comprises design of efficient processing protocols that would generate high-quality products at acceptable cost, and thus enable financial sustainability and longevous availability of those needful medicines.

Antivenom usage is not completely devoid of adverse reactions whose pathogenesis has not yet been fully understood [9]. For now, such effects have been attributed to the properties of the therapeutic itself (total amount of proteins, purity, specific activity, aggregate content, form of active drug, and formulation) and/or to some extent also to heterologous nature of animal IgGs compared to humans [9,10]. Still, most of these quality-related features are a result of the production procedure itself.

The whole IgGs are extracted from animal plasma, mostly equine or ovine, by various refinement strategies and either serve as the intermediate in further processing towards F(ab’)_2_ or Fab fragments or represent the final form of the active drug. The well-established and most commonly used industrial purification procedures on the large-scale are salting-out of IgGs with sodium or ammonium sulphate (ASP) and caprylic acid (CA) precipitation of non-IgG proteins [11,12]. The reported limitations of salt-mediated methods are not only the low recovery of antibody activity (less than 50%) [10] and the poor yield, but also the hardly reachable compliance with regulatory requirements concerning purity [11] and aggregate content [13,14]. CA as an alternative [14,15] successfully circumvents these shortcomings, yielding aggregate-free preparations [10]. It is believed that the phenomenon is associated with the ability of CA to precipitate only unwanted plasma proteins while leaving IgGs permanently in a soluble form, therefore preventing structural changes, which have been recognized as aggregation triggers in studies on human IgGs [16,17,18].

Chromatography-based principles have been utilized for IgG-based antivenom production to an increasing degree at laboratory and manufacturing scale. Protein A/G affinity chromatography (AC) currently represents the industrial gold standard of monoclonal antibodies extraction due to its high selectivity and robust performance [19,20]. Additionally, there are attempts for its implementation into antitoxin processing [21]. However, according to the recent research from the field, adsorption to the stationary phase and exposure to low pH conditions during the elution step can cause transient but extensive conformational changes and structural instability of IgGs, besides the formation of aggregates after secondary stress exposure [18]. Furthermore, due to the resin expensiveness, its limited loading capacity [20,22] and significant decrease in neutralizing activity of the final product [10], AC has been considered as cost-ineffective approach, and is therefore especially unaffordable for patients from low-income countries [10,23]. Ion-exchange chromatography (IEX) has been recognized as a method of choice for IgG and its fragments polishing after the initial fractionation step involving enzyme digestion, salting-out, or caprylic acid precipitation [22,24,25]. The anion-exchange approach (AEX) is preferred, since contaminating proteins bind to the AEX material and IgGs remain in the flow-through fraction [24,26] preserving their conformational and/or structural stability. Alternatively, in cation-exchange chromatography (CEX) IgGs bind, while contaminants pass through the column unhindered. Recently, unconventional behavior of the target protein during binding and elution has been described [27,28], which led to the hypothesis that this principle destabilizes protein structure and causes aggregate formation [16].

Since the above-described chromatographic methods employ different separation principles for purification, all of them very likely affect potency- and safety-related IgG properties. Distinct impurity profiles, in both quantity and type, are highly expected. Furthermore, all evaluated processing methods extract the entire IgG fraction from plasma, containing both venom-specific and therapeutically irrelevant antibodies. Therefore, such preparations represent a complex mixture of IgGs and their various subclasses whose functional and/or structural properties, as well as relative distributions, might be affected differently. Recently, it has been hypothesized that purification protocols including more aggressive treatments, such as precipitation with high salt concentration or elution at low pH, contribute to IgG denaturation which makes them more prone to aggregation [6,29]. The current findings on refinement strategy’s influence on antivenom properties originate from studies performed with plasma samples from different animal species as systems with their own specificities and IgG profiles or plasma samples of different venoms’ specificities and neutralization power. Moreover, these studies were not carried out in direct comparison, so the results gained so far are often contradictory and misleading, especially in terms of the therapeutics’ stability and efficacy. To the best of our knowledge, there is no systematic investigation that succeeded to undoubtedly elucidate the influence of the most widely practiced purification protocols on the relevant antivenom properties.

Here we aim to clarify the impact of ammonium sulphate precipitation (ASP), caprylic acid precipitation (CAP), anion-exchange (AEX) and cation-exchange chromatography (CEX), as well as protein A affinity chromatography (AC) on the purity profile, aggregate content, and thermal stability of IgG-based antivenoms. Our objective was to link refinement strategies to the features of the final product, with special emphasis on the impact of IgG subclass distribution on in vivo protective efficacy. Fractionation was performed on the unique hyperimmune *Vipera ammodytes ammodytes* (*Vaa*)-specific plasma pool as the starting material. All final products were formulated in the same buffer and analyzed simultaneously. We believe that our findings might improve and ease the process of rational designing in novel downstream processing strategies, as well as guide the optimization of the current ones by providing information on the impact of each purification principle on the quality of the final products.

## 2. Results

### 2.1. Purity of IgG Samples

**Figure 1:**
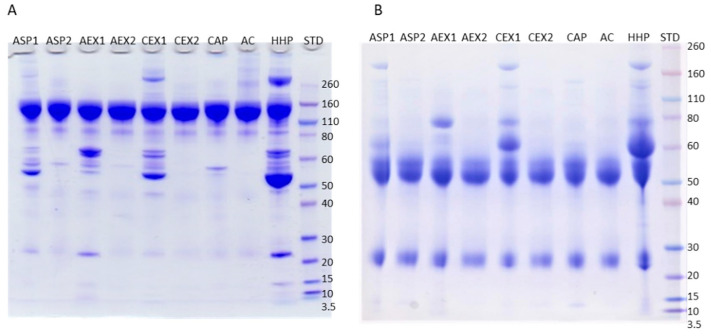
SDS-PAGE analysis of the IgG fractions from ammonium sulphate precipitation (ASP), anion-exchange chromatography (AEX), cation-exchange chromatography (CEX), caprylic acid precipitation (CAP) and protein A affinity chromatography (AC) refinement protocols, performed under non-reducing (**A**) and reducing (**B**) conditions using MOPS buffer on 4–12% Bis-Tris gel. The amount of 20 μg of protein was loaded per lane and the gel was stained with CBB R250. Samples denoted with “2” were additionally purified by caprylic acid precipitation step. HHP stands for hyperimmune horse plasma. The molecular weight standards (STD) are on the right side.

**Figure 2:**
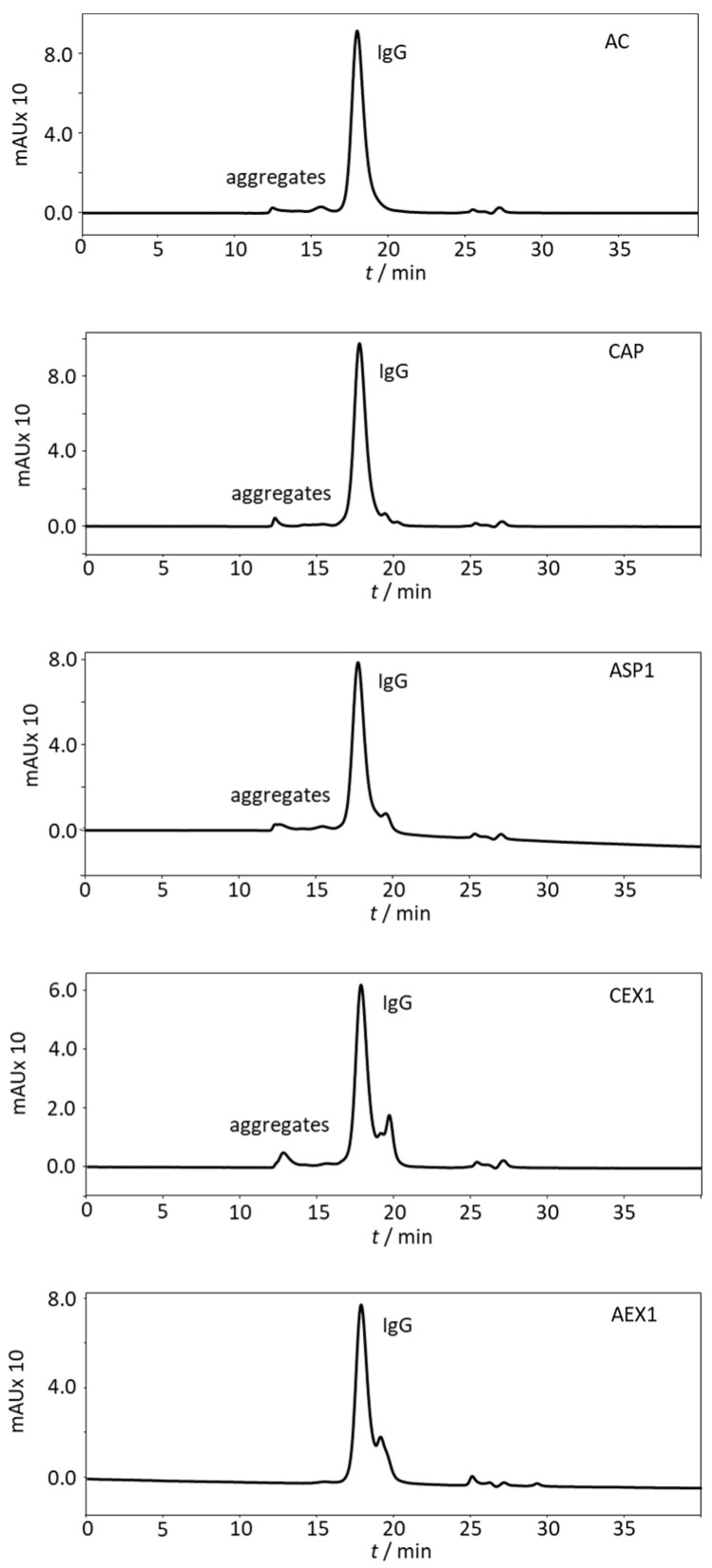
SEC-HPLC chromatograms of IgG purified fractions obtained by protein A affinity chromatography (AC), caprylic acid precipitation (CAP), ammonium sulphate precipitation (ASP1), cation-exchange chromatography (CEX1) and anion-exchange chromatography (AEX1). Analysis was performed on TSKGel G3000SWXL column (7.8 × 300 mm) with 0.1 M phosphate–sulphate running buffer, pH 6.6 at a flow rate of 0.5 mL min^−1^. The samples (1 mg mL^−1^) were pre-treated by centrifugation for particulate removal, then loaded in a volume of 50 μL. The absorbance was monitored at 280 nm.

IgG preparations derived from the unique pool of *Vaa*-specific hyperimmune horse plasma (HHP) using five different refinement protocols demonstrated differences in purity degree, as monitored by SDS-PAGE (Figure 1) and SEC-HPLC (Figure 2). Both methods were also used for IgG monomer content quantification (Table 1). Caprylic acid precipitation (CAP) and protein A affinity chromatography (AC) processing produced almost completely pure samples (above 90%) (Table 1). A single step was sufficient to achieve removal of almost all unwanted plasma proteins (Figure 1). In contrary, products obtained by ammonium sulphate precipitation (ASP1) as well as anion- and cation-exchange chromatography procedures (AEX1 and CEX1) retained noticeable amount of impurities (only 73 to 83% purity) (Figure 1; Table 1). As such, they were not suitable for the thermal stability analysis and were further purified by caprylic acid precipitation step in order to acquire satisfactory purity level. The introduction of the additional refinement step notably improved the quality of the IgG preparations (ASP2, AEX2, and CEX2 with purities above 97%) (Figure 1; Table 1). SEC-HPLC profiles (Figure 2) were in accordance with those resulting from SDS-PAGE analysis (Figure 1).

### 2.2. Impurities Identification

**Figure 3:**
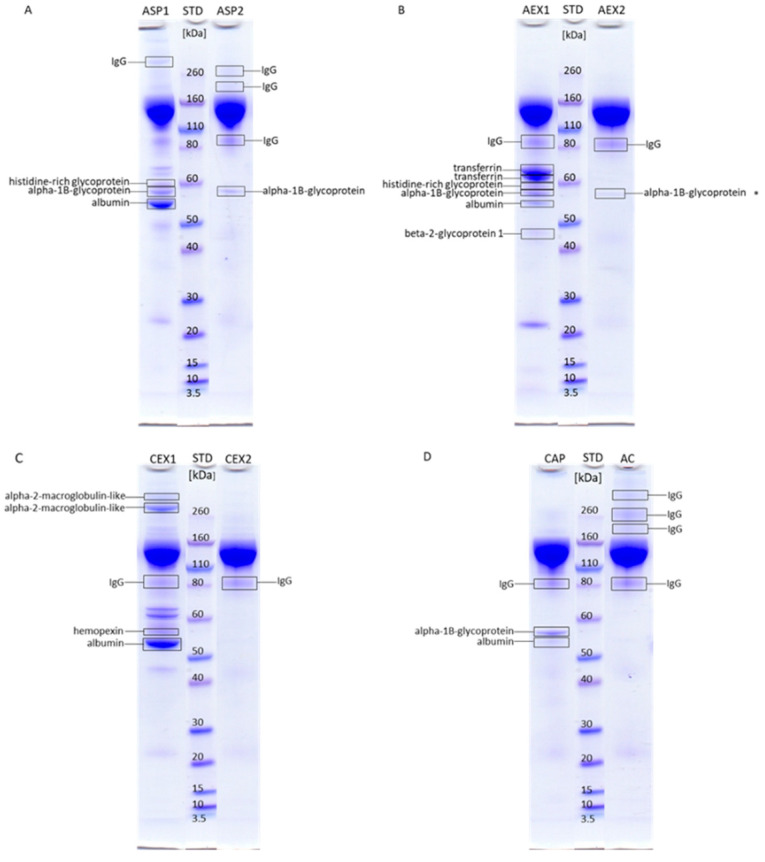
SDS-PAGE analysis of protein bands under non-reducing conditions from the purified IgG fractions obtained by ASP (**A**), AEX (**B**), CEX (**C**), CAP (**D**), and AC (**D**) refinement protocols with annotation of protein bands subjected to mass spectrometry for analysis. Samples denoted with “2” were additionally purified by caprylic acid precipitation step. The STD abbreviation stands for molecular weight standard. The list of proteins identified by MS/MS analysis is given in Supplementary Table S1 denoted by the same numbers as gel pieces on the SDS-PAGE gel in Supplementary Figure S1. Alpha-1B-glycoprotein in AEX2 fraction denoted with asterisk was identified only by peptide mass fingerprinting.

Each IgG purification procedure was associated with a specific impurity profile of the respective final product. IgG and other plasma proteins were identified by mass spectrometry (MS). Albumin was the major contaminant in IgG fractions obtained by ASP (ASP1) (Figure 3A) and CEX refinement protocols (CEX1) (Figure 3C), while it was present only in traces after AEX (AEX1) (Figure 3B) and CAP (Figure 3D). The affinity-purified IgG sample (AC) was completely devoid of albumin (Figure 3D). Among other contaminants, ASP (ASP1) and AEX fractions (AEX1) also contained histidine-rich glycoprotein and alpha-1B-glycoprotein (Figure 3A,B). AEX1 also contained beta-2-glycoprotein-1 and transferrin impurities (Figure 3B). Residual proteins in the CEX sample (CEX1) were alpha-2-macroglobulin-like protein and hemopexin (Figure 3C), while in the CAP fraction (CAP) alpha-1B-glycoprotein remained (Figure 3D). Further polishing with CA successfully removed most of the contaminants except alpha-1B-glycoprotein from ASP (ASP2) (Figure 3A) and AEX samples (AEX2) (Figure 3B).

### 2.3. IgG Thermal Stabiliy

**Figure 4:**
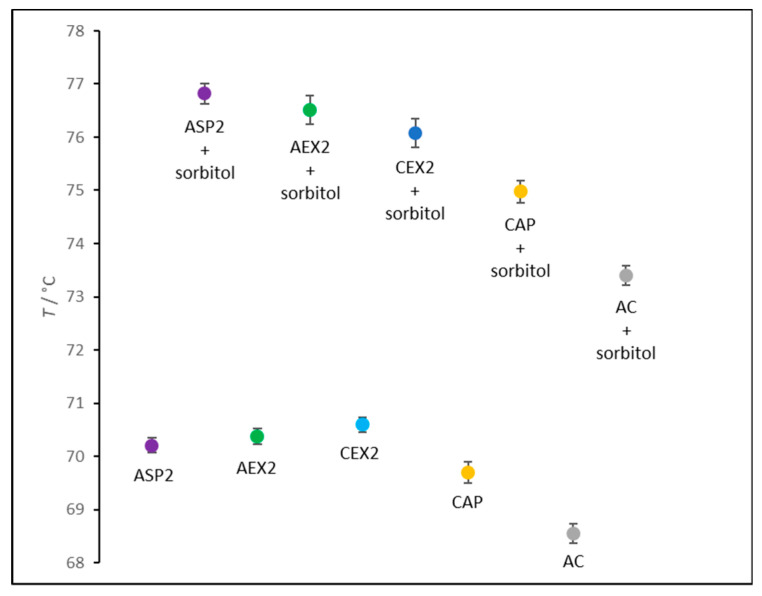
Melting temperatures (*T_m_*) of highly pure IgG preparations (m = 25 μg) from ASP, AEX, CEX, CAP, and AC protocols, with or without sorbitol (2 M) as stabilizing agent, determined by TSA. ASP2, AEX2, and CEX2 fractions were obtained after additional precipitation of residual non-IgG proteins by caprylic acid employed in final concentrations of 2%, 0.5%, and 1% (*V*/*V*), respectively. Results are given as mean of *T_m_* values ± SE (denoted by error bars) from two independently prepared samples each analyzed in pentaplicate.

The thermal stability of IgGs prepared by different refinement protocols was monitored by thermal shift assay (TSA). CAP and AC processing gave highly pure IgG preparations obtained in a single step, while ASP, AEX, and CEX samples retained noticeable amount of impurities (Figure 1; Table 1). So, the additional purification step by caprylic acid precipitation was introduced for their removal prior to stability analyses. According to the results, irrespective of the employed refinement method, all IgG preparations exhibited similar thermal stability, reaching the melting temperature (*T_m_*) of about 70 °C, with the exception of the AC-derived sample for which an approximately 2 °C lower *T_m_* was obtained (Figure 4). The addition of 2 M sorbitol improved IgG resistance to denaturation in all preparations. It resulted in *T_m_* increase of 5 to 7 °C depending on the processing strategy (Figure 4).

### 2.4. Aggregate Content

The impact of different refinement procedures on the aggregate content in IgG fractions was monitored by SEC-HPLC. As shown in Figure 2 and Table 2, only anion-exchange chromatography resulted in an aggregate-free product (AEX1). The protocol based on protein A affinity chromatography (AC) was associated with the highest degree of aggregation (above 6%), followed by cation-exchange chromatography (CEX1) (5.4%), and finally, ammonium sulphate (ASP1) (4.5%) and caprylic acid precipitation (CAP) (2.3%). In ASP1 and CEX1 aggregates were significantly reduced after caprylic acid treatment and their share decreased below 1.5% (ASP2, CEX2).

### 2.5. IgG subclass Distribution

The old nomenclature describes five horse IgG subclasses named IgGa, IgGb, IgGc, IgG(T), and IgG(B). According to the new nomenclature, implemented after identifying seven horse heavy chain constant region genes, there are seven IgG subclasses reassigned as IgG1 to IgG7 where IgGa corresponds to IgG1, IgGb to IgG4 and IgG7, IgGc to IgG6 and IgG(T) to both IgG3 and IgG5 [30]. Throughout this work, the “earlier” names of the IgG subclasses are used due to their availability on the market under the old nomenclature names.

**Figure 5:**
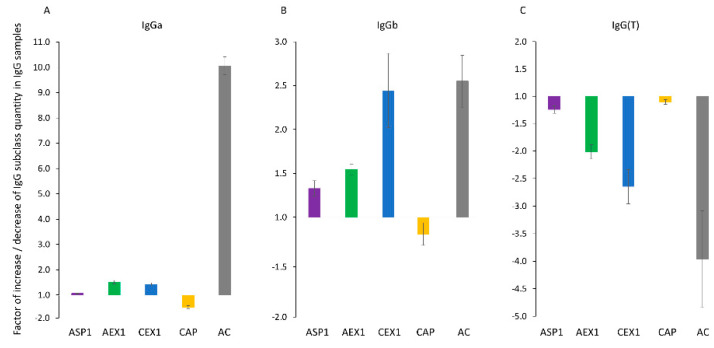
IgGa (**A**), IgGb (**B**) and IgG(T) (**C**) subclass composition changes due to the ammonium sulphate precipitation (ASP), anion-exchange chromatography (AEX), cation-exchange chromatography (CEX), caprylic acid precipitation (CAP) and protein A affinity chromatography (AC) purification procedures. Each column represents the factor of IgG subclass quantity increase (enrichment) or decrease (loss) in relation to the amount in the hyperimmune horse plasma. Its value equals in inversely proportional manner to the factor by which the IgG quantity needs to be increased or decreased in respective ELISA assay in order to yield absorbance of 1.0 at 492 nm. Results are given as mean from at least three determinations ± SE (denoted by error bars).

As determined by ELISA, the distribution of certain IgG subclasses, namely IgGa, IgGb, and IgG(T), within the final products was influenced differently. Depending on the employed purification protocol enrichment or depletion was observed (Figure 5). The results are expressed as a factor of increase or decrease of the respective IgG subclass quantity in comparison to the HHP as a starting material. Impact of protein A affinity chromatography (AC) proved to be the most prominent, since it caused significant enrichment of IgGa (Figure 5A), moderate enrichment of IgGb (Figure 5B) and substantial loss of IgG(T) subclass (Figure 5C). Cation and anion-exchange chromatography (CEX and AEX) had similar effect on the subclass distribution. Both methods caused the loss of IgG(T) subclass, although to a lesser extend in comparison to protein A affinity chromatography processing (Figure 5C), as well as moderate increase of IgGb (Figure 5B) and slight increase of IgGa content (Figure 5A) within the respective sample. Other two refinement methods did not have substantial impact on the subclass composition. The reduction of IgG(T) subclass presence in AC and CEX purified fractions was additionally confirmed by ELISA for determination of IgG(T) antibodies specific only for ammodytoxins and haemorrhagins, two most relevant groups of *Vaa* venom components involved in envenomation pathology (Supplementary Figure S2).

### 2.6. Vaa Venom-Specific Protective Efficacy

Neutralization potencies of HHP and selected IgG preparations, together with specific activities of their active drug, are summarized in Table 3. Since the CEX1 preparation had low purity (Figure 1; Table 1), the in vivo assay was performed only with its additionally refined fraction (CEX2). According to the lethal toxicity neutralization assay in mice, no loss of activity in comparison to the starting material occurred for the CAP fraction. On the contrary, in CEX2 and AC preparations the specific activity was reduced in half or more than a half, respectively.

A summary of all the results presented above is given in Supplementary Table S2.

## 3. Discussion

In the field of antivenom manufacturing there has been a constant effort to design an optimal production strategy that will put the safety and potency of these immunotherapeutics in balance with their sustainable cost-effectiveness in order to successfully fight snakebite envenoming consequences worldwide. The aim of our research was to systematically investigate the influence of five different IgG purification principles used to extract whole IgGs from *Vaa*-specific HHP on their certain quality-related properties. We have to state at this point that IgG yield was not within the scope of this investigation. In addition to IgG purity and contents of monomer and aggregates, subclass distribution changes within the IgG fractions were monitored. To the best of our knowledge, the latter has not previously been reported. Just for comparison, such analysis has already been implemented as a regulatory quality control requirement for human IgG products [31]. Further, IgG extractions were performed on analytical scale without any intention to imply that IgG antivenoms manufactured by protocols involving either of purification principles described above are neither more nor less valuable. Starting the fractionation from the unique HHP sample, storing the IgG preparations continuously under the same conditions, and conducting the experiments simultaneously enabled us to obtain results that can for the first time be presented in direct comparison.

### 3.1. Protein A Affinity Chromatography

As expected, due to its high binding specificity for the target protein, AC gave almost completely pure product (Figure 1 and Figure 2; Table 1) without any protein contamination (Figure 3D). At the same time it yielded the highest aggregate content of 6.4% (Table 2). Studies on affinity-purified IgG monoclonal antibodies already demonstrated that during elution steps at low pH transient but extensive structural changes lead to aggregate formation after secondary stress exposure [18]. Since our experimental setting was even harsher, the acidic environment during the elution step was probably in itself sufficient to provoke aggregation. Compared to IgG subclass distribution in normal equine plasma [32], AC affected their presence in the refined product the most negatively. AC provided a sample that was enriched with IgGa and IgGb subclasses but depleted from IgG(T) (Figure 5). This finding is corroborated by results for equine IgG subclasses refined by protein A affinity chromatography [32].

### 3.2. Precipitation-Based Purification Procedures

CAP, a cost-effective and simple one-step IgG refinement approach, relies on selective precipitation of the majority of plasma proteins while leaving IgGs in solution [33]. Although unspecific, this method gave a product of almost equal IgG monomer content as the highly specific AC (Figure 2; Table 1), while also being the second best product concerning aggregate content (only 2.3%) (Table 2). We previously showed that CA in concentrations ranging from 1 to 3% does not precipitate IgGs although the lowest concentration significantly impaired their purity [6]. Already 3% CA precipitates fraction of IgGs while higher concentrations have an unfavorable impact on the supernatant showing excessive turbidity [6,10]. Since the IgG yield was not focus of this work, plasma fractionation was performed with 3% CA as a compromise between the highest possible purity of IgG and its minimal loss. Two protein contaminants were identified by MS analysis: albumin, remaining just in traces, and alpha-1B-glycoprotein (Figure 3D). We believe that the former could have been completely eliminated if the precipitation was performed with higher CA concentration, which would probably adversely affect other sample properties such as turbidity, as mentioned above. The main putative mechanism of CA action is based on its hydrophobicity that enables precipitation of acidic proteins, while basic ones, like IgGs, are protected by charge mediated hydration, therefore remaining in the solution [33,34]. According to MS results, alpha-1B-glycoprotein, although slightly acidic, exhibited resistance to the impact of CA (Figure 3D). This implies that the outcome of CAP under given experimental conditions could possibly be influenced by other factors as well. Although the share of aggregates in CAP-purified samples was low, 2.3% (Table 2), their presence indicated that CAP does not completely prevent aggregation. Similarly, increased levels of aggregation were noticed in some preparations of affinity-purified monoclonal antibodies following CA polishing treatment [34]. It should be emphasized that the CAP purification work-flow did not induce IgG subclass redistribution (Figure 5), as already reported by Halassy et al. [35], which is especially important with regard to the IgG(T) isotype as the most important one for the toxin neutralization activity that will be discussed in more detail later. ASP, as another precipitation procedure, also showed no significant impact on IgG subclass composition (Figure 5). However, this method exhibited poor IgG extraction capability by yielding preparation with noticeable amount of impurities (Figure 1; Table 1) and aggregates (Table 2), as already reported in the literature [11,14,21,36].

### 3.3. Ion-Exchange Chromatography

IEX is a very versatile method as it can be modulated by changing pH and/or ionic strength of the mobile phase. Whether the molecules of interest will pass the column freely or bind to the functional groups and elute subsequently depends on their isoelectric point, as well as the pH value and ionic strength of the buffer systems. For both, AEX and CEX, we optimized operational conditions in a manner to mostly separate IgGs from albumin, the dominant plasma protein. Horse serum proteome analysis by 2D-PAGE revealed that the pI value range of albumin (5.3–6.0) and IgG (5.8–7.0) overlaps considerably [37,38]. Hence, it was not expected that either of two IEX modes would be efficient enough to provide pure IgG fractions in one step. To our surprise, the IgG purity in the AEX and CEX preparations was quite high and comparable to the IgG fraction prepared by ASP (Table 1). The latter is still the most commonly used antivenom production procedure today. Such high levels of purity could be explained by the fact that most plasma proteins are acidic and separate well from the more basic IgGs under the applied IEX conditions. The impurity profiles of both preparations were similar in regard to the most abundant contaminating proteins: albumin and transferrin (Figure 3B,C). While albumin was dominantly present in CEX-based sample (Figure 3C), transferrin was the major residual protein in preparation obtained by AEX (Figure 3B). Despite of unsuccessful attempts to identify some impurities by MS, we strongly believe that retention of transferrin also occurred for CEX sample. This assumption is based on the fact that the positions of two distinctive but faint, unidentified protein bands in SDS-PAGE correspond to bands in the AEX-based sample (Figure 3B) which were identified to be transferrin. In contrast to the sample obtained by AEX, the CEX preparation also contained alpha-2-macroglobulin-like protein (Figure 3C). Although alpha-1B-glycoprotein is mildly acidic protein [38] and was expected to bind to AEX support, it has emerged in an unbound fraction (Figure 3B) which implies that the net charge is not a sole factor influencing the chromatographic behavior, as already observed [39]. Positively charged patches formed on the surface of alpha-1B-glycoprotein might prevail the protein net charge, preventing it from adsorption to the stationary phase. In the samples from both AEX and CEX we observed the loss of IgG(T) subclass molecules (Figure 5C) which is in accordance with the fact that they exhibit the lowest pI values of all IgG subclasses [37]. In contrary to AEX-based preparation and similar to that obtained by AC, the CEX sample has also been highly enriched with aggregates (5.4%) (Table 2). According to the data in the literature, during the salt-step elution a pH drop occurs as a consequence of ongoing competitive equilibrium between salt ions and H^+^/OH^−^ ions [40,41]. The cations in the elution buffer that contain high salt concentration displace H^+^ ions in the stationary phase and enter the mobile phase causing temporary pH lowering. Such transient states involving high salt concentration and low pH lead to protein denaturation and aggregate formation, presence of which is associated with the appearance of multi-peak elution profile [16]. Although IgGs are more resistant to environmental changes than some other proteins [42], it seems that consecutive exposure to unfavorable process-related conditions strongly affects their stability.

### 3.4. Thermal Stability

**Figure 6:**
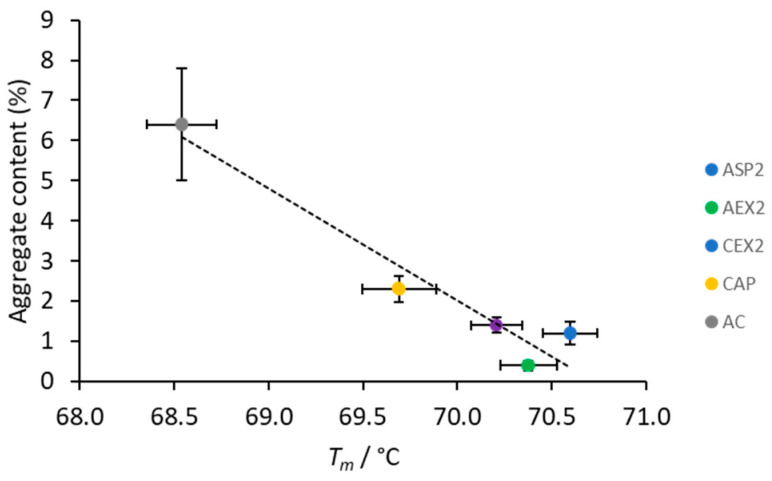
Correlation between *T_m_* values of IgG molecules and aggregate content in purified fractions obtained by ammonium sulphate precipitation (ASP), anion-exchange chromatography (AEX), cation-exchange chromatography (CEX), caprylic acid precipitation (CAP) and protein A affinity chromatography (AC). Thermal stability results are given as mean of *T_m_* values ± SE (denoted by error bars) from two independently prepared samples each analyzed in pentaplicate. Aggregate content results are given as mean of four analysis ± SE (denoted by error bars).

Thermal stability analyses by TSA required the samples of the highest purity. Since ASP1, AEX1, and CEX1 preparations were substantially contaminated (Figure 1; Table 1), an additional CAP step was introduced to further extract IgGs (Table 1) without affecting subclass composition. CA concentrations were adjusted according to the quantity of the contaminating proteins for their depletion, at the same time preserving the IgG yield. CA-mediated polishing resulted in substantially higher purity and lower aggregate share in ASP2, AEX2 and CEX2 samples (Table 1 and Table 2), suggesting that CAP might be an aggregate reducing procedure. ASP2, AEX2 and CEX2 samples had higher *T_m_* values and lower aggregate content in comparison to the CAP and AC fractions that contained increased amount of aggregates and reduced *T_m_* values (Figure 4; Table 2). Accordingly, we found a high negative correlation between thermal stability of IgG molecules with the aggregate content in the preparations (Figure 6).

Aggregated IgG molecules are presumably more prone to denaturation, significantly affecting the melting temperature of the sample. This is opposite to our previous finding where no correlation was found between the thermal stability and aggregate content of the analyzed proteins [43]. The possible explanation might lie in the fact that determination of IgGs *T_m_* values in all tested preparations was performed in the same matrix in contrast to the reported study. Furthermore, notably lower *T_m_* value of the AC-purified IgGs might be linked not only to the highest aggregate content, but also to the most pronounced changes in the subclass distribution (Figure 5). The substantial loss of IgG(T) subclass might contribute to the decreased thermal stability, if one presumes that horse IgG subclasses differ in *T_m_* values, as has already been shown for human IgG subclasses [17]. Regardless of the employed purification method, sorbitol has once again been confirmed as safe and effective in stabilizing equine IgG preparations [44], as it increases the melting temperature by as much as 5–7 °C (Figure 4).

### 3.5. IgG Subclass Redistribution in Relation to Venom-Neutralising Activity

We would like to particularly emphasize our results related to the *Vaa* venom-specific IgGa, IgGb and IgG(T) subclass distribution changes (Figure 5), which imply that different IgG purification procedures affect their relative proportions in the final product. Namely, as was already mentioned, AC caused the highest alteration of the IgG subclass composition, generating a sample with notably enriched IgGa, moderately enriched IgGb and the considerable loss of IgG(T) (Figure 5). The CEX- and AEX-based samples underwent subclass rearrangement with similar tendency except for the insignificant enrichment of IgGa (Figure 5). IgG(T) subclass is generated in large amounts in equine plasma during the hyperimmunization process [45] and its prevailing role in the toxin neutralization activity has been described [45,46,47,48]. Accordingly, we demonstrated that AC and CEX preparations, which suffered the most prominent IgG(T) loss, had a specific neutralization activity towards *Vaa* venom reduced by more than 50% (Table 3). Such reduction of toxin-neutralizing potential by AC has already been reported [49], and here we demonstrated that this is due to the IgG(T) subclass loss. As expected, CAP preparations had the same specific activity as the starting plasma material (Table 3). Such result is in accordance with the one obtained for another horse plasma pool of identical venom specificity [6].

## 4. Conclusions

In this comparative work, the influence of five different IgG purification concepts on the type and amount of impurities, in addition to aggregate content in the final preparations, was studied for the first time. The particularly valuable finding is related to the influence of each refinement protocol on the IgG subclass distribution. We have demonstrated that the loss of IgG(T) subclass within the purified sample is the primary cause of the significant reduction of its venom-neutralizing potential. Of all the methods tested, caprylic acid purification principle was the one that stood out as economically-accessible and easily-feasible procedure with high IgG extraction power that generates a sample of low aggregate content and unaltered IgG subclass composition. Anion-exchange chromatography is particularly interesting due to its restrain of aggregation and a great potential for final polishing, but it has to be performed at pH values more distant (lower) from pI values of IgGs. Otherwise it could induce IgG subclass redistribution and unwanted loss of IgG(T) subclass. Our results provide evidence that, alongside the presence of aggregates and impurities in the refined sample, possible changes within IgG subclass distribution and their influence on the antivenom neutralization activity, should be undoubtedly taken into consideration when (re)designing downstream processing protocol.

## 5. Materials and Methods

### 5.1. Animals, Snake Venom, Plasma Pool, and Reagents

The adult mice (strain NIH Ola/Hsd, both sexes, 18–20 g) for lethal toxicity neutralization assay were purchased from the Institute of Immunology Inc., Zagreb, Croatia. The procedure was approved by the Croatian Ministry of Agriculture, Veterinary and Food Safety Directorate (UP/I-322-01/17-01/75, permission no. 525-10/0255-17-16, date 12 December 2017) and the University of Zagreb’s Animal Welfare Committee. The approval is based on the positive opinion of the National Ethical Committee (EP 110/2017). Animal work was in accordance to Croatian Law on Animal Welfare (2017) which complies strictly with the EC Directive and the ARRIVE guideline for the Report for in vivo experiments [50]. Mice were housed under a 12-h light/12-h dark cycle and at constant temperature of 22 °C. A standard diet (Mucedola srl., Settimo Milanese MI, Italy) and water were supplied ad libidum during the entire duration of the experiments. The animals were monitored for any signs of pain, suffering and distress associated with the procedure.

Crude venom of *V. ammodytes ammodytes* (*Vaa*) and a pool of *Vaa*-specific hyperimmune horse plasma were provided by the Institute of Immunology Inc., Zagreb, Croatia.

Caprylic acid (≥98%), *o*-phenylenediamine dihydrochloride (OPD), bovine serum albumin (BSA), *α*-cyano-4-hydroxycinnamic acid (HCCA), Coomassie Brilliant Blue (CBB) R250, dithiothreitol (DTT), iodoacetamide (IAA), and trifluoroacetic acid (TFA) were from Sigma-Aldrich, USA. Sorbitol, acetonitrile (ACN) and Tris base were from Merck, Germany. 3-(n-morpholino)propanesulphonic acid (MOPS) and 2-(n-morpholino)ethanesulphonic acid (MES) monohydrate were from AppliChem, Germany. Novex Sharp Pre-Stained Protein Standard was from Invitrogen, Waltham, MA, USA. Trypsin was from Roche, Germany. Mouse anti-horse IgGa (MCA1902GA; IgG1 isotype) and mouse anti-horse IgGb (MCA1901GA; isotype IgG1) were from Bio-Rad Laboratories, USA. HRP conjugated goat anti-mouse IgG was from Organon Teknika, Cappel Division, USA. HRP-conjugated goat anti-horse IgG(T) (ab112875) was from Abcam, UK. Other chemicals for buffers and solutions were from Kemika, Croatia, unless stated otherwise.

### 5.2. IgG Purification Procedures

The unique sample of *Vaa*-specific hyperimmune horse plasma, the starting material for all purification procedures, was thermally treated for 1 h at 56 °C, followed by centrifugation at 3200× *g* for 45 min at room temperature (RT). The pellet was discarded and supernatant termed HHP was subjected to the respective fractionation process.

#### 5.2.1. Caprylic Acid Precipitation

Caprylic acid (CA) precipitation of non-IgG plasma proteins was performed according to [6] with minor modifications. CA was added to two-fold diluted HHP with saline in a dropwise manner until the final concentration of 3% (*V/V*) was reached. Reaction mixture (*V* = 6 mL) was vigorous stirred (750 rpm) for 1 h at 23 °C in a thermomixer (Eppendorf, Germany), followed by centrifugation (2800× *g* for 45 min). The obtained IgG-enriched supernatant was filtered through a cellulose acetate filter with a pore size of 5 μm (Sartorius, Göttingen, Germany).

#### 5.2.2. Ammonium Sulphate Precipitation

For salting-out of IgGs with ammonium sulphate (ASP) the protocol described by Rojas et al. [14] was implemented, with some modifications. HHP was mixed with an equal volume of 24% (*m*/*V*) ammonium sulphate solution and first stirred vigorously (800 rpm) for 1 h at 23 °C in a thermomixer and then left unstirred overnight at RT. The volume of reaction mixture was 6 mL. After centrifugation (3000× *g* for 30 min), the pellet was discarded, while crude ammonium sulphate was added to the supernatant until its final concentration was 24% (*m/V*). Following another incubation under conditions from the first precipitation step and the supernatant removal, the IgG-enriched precipitate was dissolved in 50 mM MES, pH 5.5 (*V* = 5 mL).

#### 5.2.3. Anion-Exchange Chromatography

Anion-exchange chromatography (AEX) was performed in a batch mode following conditions giving the purest IgG fraction, according to the results from our previous study (data presented at Annual Meeting of the Croatian Immunological Society, HID 2017, Zagreb, Croatia). HHP, 5-fold diluted with 25 mM Tris/HCl + 35 mM NaCl binding buffer, pH 8.0 was incubated with Toyopearl SuperQ-650S stationary phase (0.5 mL sample per 1.2 mL matrix; Tosoh Bioscience, Tokyo, Japan) in a thermomixer (800 rpm) for 1 h at 23 °C. Following a short spin centrifugation, the IgG-enriched supernatant as the final product was collected.

#### 5.2.4. Cation-Exchange Chromatography

In cation-exchange chromatography (CEX) HHP, 10-fold diluted with 20 mM MES binding buffer, pH 6.0 was loaded to the CIMultus SO3-1 monolith (*V* = 80 mL; BIA Separations, Slovenia) (*V* = 130–150 mL/run) at a flow rate of 5 mL min^−1^. The bound fraction was eluted from the column with 1 M NaCl in the binding buffer, yielding IgG-based preparation.

#### 5.2.5. Protein A Affinity Chromatography

Protein A affinity chromatography (AC) was performed by applying 2-fold diluted HHP (7 mL/run) on the MabSelect column (*V* = 2 × 1 mL; GE Healthcare, USA) with 20 mM Tris/HCl binding buffer, pH 7.4 at a flow rate of 1 mL min^−1^. The bound antibodies were eluted with 20 mM citric acid, pH 2.5 at a flow rate of 2 mL min^−1^ and mixed with 1 M Tris, pH 10.0 to achieve neutral pH.

### 5.3. Diafiltration

IgG preparations obtained from all purification procedures were diafiltrated using Vivaspin device (Sartorius, Germany) with a 100 kDa molecular weight cut-off (MWCO) polyethersulfone membrane. Samples from ASP and CEX processing were first desalted by diafiltration into 50 mM MES buffer, pH 5.5. All final products, including those of lower purity that were submitted to additional caprylic acid treatment step, were diafiltrated into 0.2 M phosphate buffer, pH 6.0, ensuring matrix uniformity prior further analysis. In each diafiltration step the buffer exchange factor was approximately 8000.

### 5.4. Thermal Shift Assay

Thermal stability of IgGs purified by different refinement protocols was measured by thermal shift assay (TSA). Prior its performance, the final products of lower purity were additionally processed for caprylic acid-mediated removal of remaining impurities, as described in Section 5.2.1. Concentration of precipitating agent was optimized depending on the contamination degree, ranging from 0.5 to 2% (*V/V*).

The reaction mixtures were prepared in optical tubes (Applied Biosystems, Waltham, MA, USA) by mixing 25 μL of highly pure IgG samples (1 mg mL^−1^), previously diafiltrated into 0.2 M phosphate buffer, pH 6.0, 20 μL of sorbitol (4.4 M) and 5 μL of Sypro Orange dye solution (10× concentrate; Molecular Probes, Eugene, OR, USA). In another, simultaneously performed experimental setup, sorbitol was replaced with an equal volume of 0.2 M phosphate buffer, pH 6.0. Negative controls without protein samples were also included. TSA analysis was performed in qPCR instrument (7500 Real Time PCR, Applied Biosystems, Waltham, MA, USA). The reaction mixtures were incubated for 10 min at 5 °C, followed by an increase in temperature at the heating rate of 1 °C min^−1^ until reaching 94 °C. The curves and the *T_m_* values were determined by nonlinear regression using GraphPad Prism software (version 5.00 for Windows, GraphPad Software, San Diego, CA, USA, www.graphpad.com). All measurements were performed in pentaplicates.

### 5.5. Purity and Aggregate Content Profiling

Size-exclusion chromatography (SEC), which was employed for monitoring of IgG purity as well as the estimation of aggregate content, was performed on TSKGel G3000SWXL column (7.8 × 300 mm; Tosoh Bioscience, Japan) in 0.1 M phosphate–sulphate running buffer, pH 6.6 at a flow rate of 0.5 mL min^−1^ and at RT on a Shimadzu HPLC system (Shimadzu, Japan). The sample (1 mg mL^−1^), pre-treated by centrifugation for particulate removal, was loaded in a volume of 50 μL. The absorbance was monitored at 280 nm. Standard proteins used for molecular weight determination were thyroglobulin (*M*_r_ 669,000), γ-globulin (*M*_r_ 150,000), ovalbumin (*M*_r_ 43,000), and ribonuclease A (*M*_r_ 13,700) from Sigma-Aldrich, St. Louis, MO, USA.

The purity of each IgG preparation (20 μg/well), obtained by the respective purification procedures, was also examined by SDS-PAGE on a 4–12% Bis-Tris gel with MOPS-based running buffer under reducing and non-reducing conditions in an Xcell SureLock Mini-Cell, according to the manufacturer’s instructions (Invitrogen, USA). CBB R250-stained protein spots were used as starting material for mass spectrometry (MS) analysis.

### 5.6. Mass Spectrometry for Protein Identification

Protein bands from IgG preparations obtained by SDS-PAGE were excised and subjected to in-gel digestion by trypsin, as follows. Gel pieces were washed three times with ultra-high quality (UHQ, conductivity < 18MO hmcm^−1^) water, then three times with UHQ water/ACN in 1:1 ratio (*V*/*V*) and once in ACN only. After ACN removal and incubation, first with NH_4_HCO_3_ (5 min), then with the same volume of ACN (15 min), gel pieces were dried under vacuum, reduced with 10 mM DTT (45 min at 56 °C) and alkylated with 54 mM IAA (30 min at RT in the dark), both prepared in 100 mM NH_4_HCO_3._ After removal of alkylation solution the gel pieces were incubated for 5 min with 100 mM NH_4_HCO_3._ The same volume of ACN was added (100 mM NH_4_HCO_3/_CAN, 1:1) followed by another incubation for 15 min. After solvent removal, the gel pieces were dried under vacuum and rehydrated in 1–20 μL of porcine trypsin solution (Roche, Germany) (10 ng of trypsin per estimated 1 μg of protein) for 45 min. Digestion was conducted in 95% 50 mM NH_4_HCO_3_ and 5% ACN (*V*/*V*) in a thermomixer (1000 rpm) overnight at 37 °C. Subsequently, 50 mM NH_4_HCO_3_ was added in each reaction tube and after 15 min-long incubation at RT, the same volume of ACN was added. After another 15 min, the supernatant was extracted, and peptides were recovered. Peptide extraction was repeated twice with 1% HCOOH/ACN 1:1 (*V*/*V*). Obtained extracts were pooled, purified by Zip-Tip C_18_ (Millipore, USA), dried, then dissolved in 0.1% TFA/ACN 1:1 (*V*/*V*) and mixed with MALDI matrix (HCCA) prepared in the same solution (0.1% TFA/ACN 1:1 (*V*/*V*); 3 mg/mL). Volume of 1 μL of each peptide sample was spotted on stainless steel MALDI target (Bruker, Germany) and measured on an ultrafleXtreme instrument (Bruker, Germany) in a positive reflectron ion mode. MS/MS spectra for peptide sequencing were measured after isolation of the monoisotopic peak. Before each measurement and after every 2–3 measured samples, the instrument was calibrated with Peptide Calibration Mix4 (LaserBio Labs, Valbonne, France), prepared with 0.1% TFA/ACN 1:1 (*V*/*V*). Obtained spectra were processed using FlexAnalysis (3.4.76.0) and BioTools (3.2.SR). Identification searches were performed against NCBI database “Other Mammalia” (05/2019 with 152,462,470 sequences) (www.matrixscience.com) and against a contaminant database using Mascot on an in-house server. Following parameters were used: precursor ion mass tolerance ±200 ppm, one missed cleavage, carbamidomethylation of Cys as a fixed modification. The variable modifications were N-terminal protein acetylation, oxidation of His, Trp, and Met and deamidation of Asn and Gln. Proteins were confidently identified by peptide mass fingerprint (PMF) and peptide sequencing scores if statistical scores were above respective threshold levels.

### 5.7. Protein and IgG Content Determination

Total protein concentration in all final products and their CA-treated fractions of enhanced purity was measured spectrophotometrically by using the Equation (1) [51].

*γ* [mg/mL] = (*A*_228.5 nm_ − *A*_234.5 nm_) × dilution factor × *f* (1)

where Ehresmann’s factor *f*, for equine IgG, a dominant molecule in each sample, of 0.2553 was used [35].

The IgG concentration in purified fractions was calculated as (SEC-determined purity in percentage/100%) × *γ* (protein)), where total protein concentration was determined according to Equation (1). Alternatively, densitometric analysis was employed. A highly pure IgG sample, prepared by protein A affinity chromatography and precisely quantified, was separated using 4–12% Bis-Tris gel in a suitable range of known quantities, together with less pure preparations. SDS-PAGE analysis was performed under conditions described in Section 5.5. The CBB R250-stained image was recorded in trans-illumination mode with Amersham Imager 680 (GE Healthcare, Chicago, IL, USA) and processed by background subtraction using the rolling ball algorithm. Following measurement of the optical density of bands detected at 150 kDa, the integrated intensity values obtained for each standard to the known amount loaded per well (in μg) gave calibration curve that was used for IgG quantification in purified fractions processed by other refinement strategies. The densitometric results corresponded to the SEC-determined concentrations.

### 5.8. IgG Subclass Quantity Determination

The *Vaa* venom-specific IgGa, IgGb and IgG(T) subclass distribution within HHP and final products was determined by the respective in-house ELISA assays, as already described [35]. Absorbance to total IgG concentration of tested samples, precisely determined by SEC and densitometry, gave curves, described either by 2nd polynom or natural logarithm, which were used for determination of IgG quantity giving *A*_492 nm_ of 1.0 value in each subclass ELISA.

### 5.9. Lethal Toxicity Neutralization Assay

The potential of HHP and IgG preparations obtained by caprylic acid precipitation, affinity, and cation-exchange chromatography to neutralize the venom’s lethal toxicity was determined by the lethal toxicity neutralization assay in mice as previously described [52]. The lethal toxicity neutralization potency (*R*) was expressed as the number of LD_50_ venom doses that can be neutralized by 1 mL of undiluted sample and calculated by Equation (2)

*R* = (Tv − 1)/ED_50_, (2)

where Tv represents the number of LD_50_ venom doses inoculated per mouse. The *R*-value was used as a measure of the protective efficacy of each sample. The specific activity (LD_50_ mg^−1^) was calculated as a ratio of the obtained *R*-value and the respective IgG concentration.

### 5.10. Data Analysis

The results of each analysis are expressed as the average (arithmetic mean) of *n* measurements ± standard error (SE). Number of measurements for each analysis (*n*) is given. Correlation between the set of different assays, expressed as *r* value, was calculated using the software Statistica 13.5 (StatSoft, TIBCO Software Inc., Palo Alto, CA, USA) with uncertainty of measurements expressed as 95% confidence interval.

## Figures and Tables

**Table 1:** SEC-HPLC-determined IgG monomer content (purity in %) in samples obtained by ammonium sulphate precipitation (ASP), anion-exchange chromatography (AEX), cation-exchange chromatography (CEX), caprylic acid precipitation (CAP) and protein A affinity chromatography (AC) refinement protocols. Samples in italic denoted with “2” were additionally purified by caprylic acid precipitation step. Results are given as mean from *n* determinations ± standard error (SE).

Purification Method	IgG Purity (%)
ASP1	83.1 ± 1.5 (*n* = 2)
*ASP2*	*96.6 ± 1.2 (n = 4)*
AEX1	76.7 ± 0.8 (*n* = 2)
*AEX2*	*98.0 ± 0.8 (n = 4)*
CEX1	72.6 ± 2.2 (*n* = 3)
*CEX2*	*97.8 ± 0.8 (n = 4)*
CAP	93.3 ± 1.4 (*n* = 4)
AC	92.1 ± 1.7 (*n* = 4)

**Table 2:** SEC-HPLC analysis of aggregates in IgG preparations obtained by ASP, AEX, CEX, CAP and AC purification protocols. Samples in italic and denoted with “2” were additionally purified by a CAP step. Results are given as mean from 4 determinations ± SE.

Purification Method	Aggregate Content (%)
ASP1	4.5 ± 1.4
*ASP2*	*1.4 ± 0.2*
AEX1	0.0 ± 0.0
*AEX2*	*0.4 ± 0.1*
CEX1	5.4 ± 0.9
*CEX2*	*1.2 ± 0.3*
CAP	2.3 ± 0.3
AC	6.4 ± 1.4

**Table 3:** In vivo neutralization potencies of hyperimmune horse plasma, CEX2, AC, and CAP IgG samples with specific activities of their active drug (IgG). Results are expressed as mean from two independently performed experiments with CEX2 and AC samples.

	Hyperimmune Horse Plasma	CEX2	AC	CAP
*R*/LD_50_ mL^−1^	23.89	14.15	<5.48	12.05
*γ*(IgG)/mg mL^−1^	22.89	31.40	13.35	12.84
Specific activity [*R*/*γ*(IgG)]	1.03	0.45	<0.41	1.07

## Supplementary Materials

The following are available online at https://www.mdpi.com/2072-6651/12/12/798/s1, Figure S1. SDS-PAGE analysis of purified IgG fractions under non-reducing conditions with annotation of protein bands identified by MS/MS analysis (in Table S1), Figure S2. Ammodytoxin- and hemorrhagin-specific IgG(T) subclass determination in purified IgG fractions, Table S1. List of proteins identified by MS/MS analysis in the IgG preparations after ASP, AEX, CEX, CAP, and AC purification processing, Table S2. Summary of quality-related properties of IgGs purified by ammonium sulphate precipitation (ASP), anion-exchange chromatography (AEX), cation-exchange chromatography (CEX), caprylic acid precipitation (CAP), and protein A affinity chromatography (AC).
