# Supplementary material for: Quality-Related Properties of Equine Immunoglobulins Purified by Different Approaches
Source: Toxins (Basel). 2020 Dec 14;12(12):798. doi: 10.3390/toxins12120798 (PMC7764988; doi:10.3390/toxins12120798)
Supplement: Supplementary file 1 [file toxins-12-00798-s001.pdf]

Supplementary Materials: Quality-Related Properties of Equine Immunoglobulins Purified by Different Approaches

Sanja Mateljak Lukačević, Tihana Kurtović, Maja Lang Balija, Marija Brgles, Stephanie Steinberger, Martina Marchetti-Deschmann and Beata Halassy

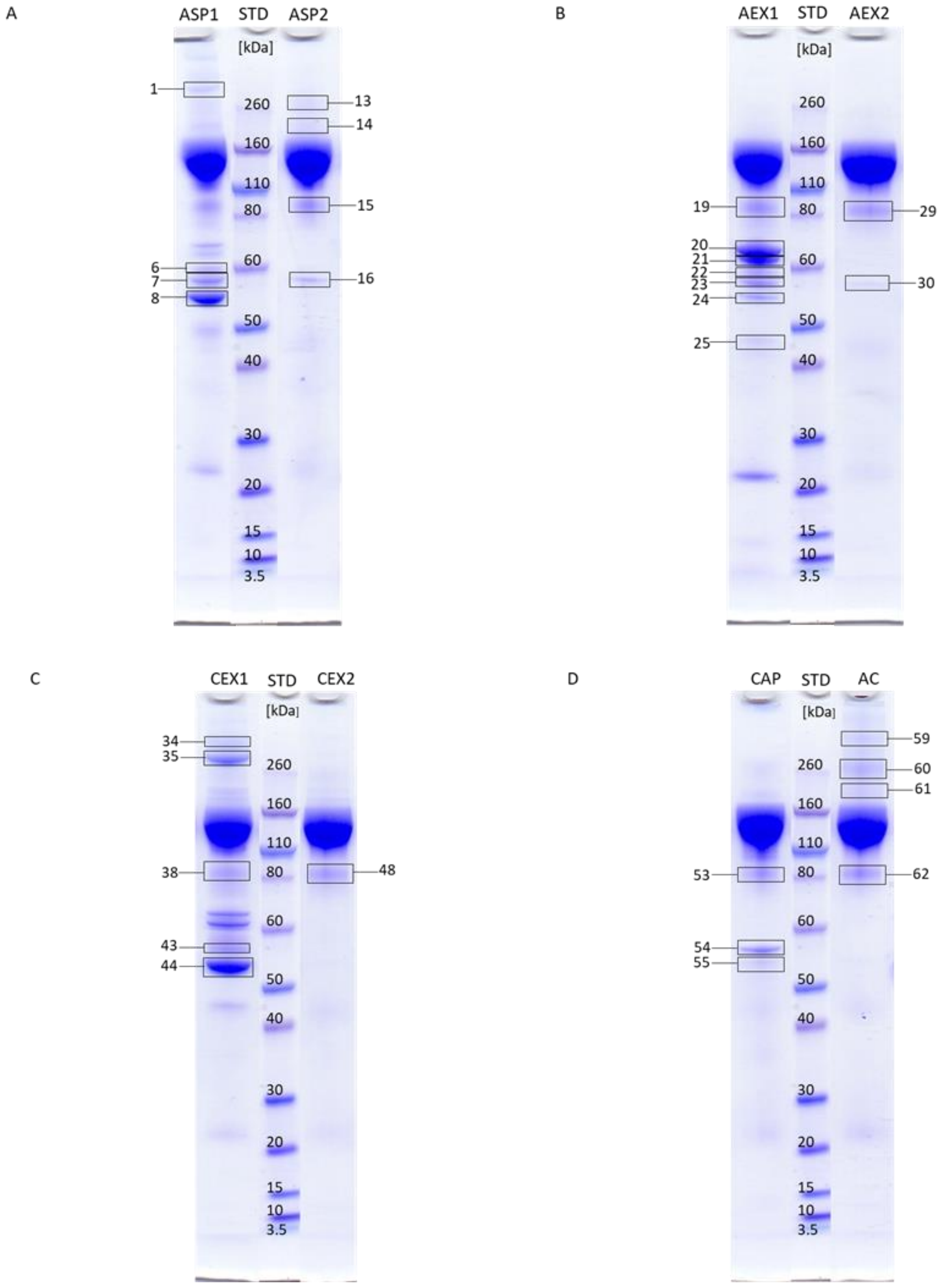

Figure S1. SDS-PAGE analysis of purified IgG fractions under non-reducing conditions with annotation of protein bands identified by MS/MS analysis (in Table S1). IgG samples were obtained by ASP (**A**), AEX (**B**), CEX (**C**), CAP (**D**) and AC (**D**) refinement protocols (same samples as in Figure 3). Other protein bands remained unidentified.

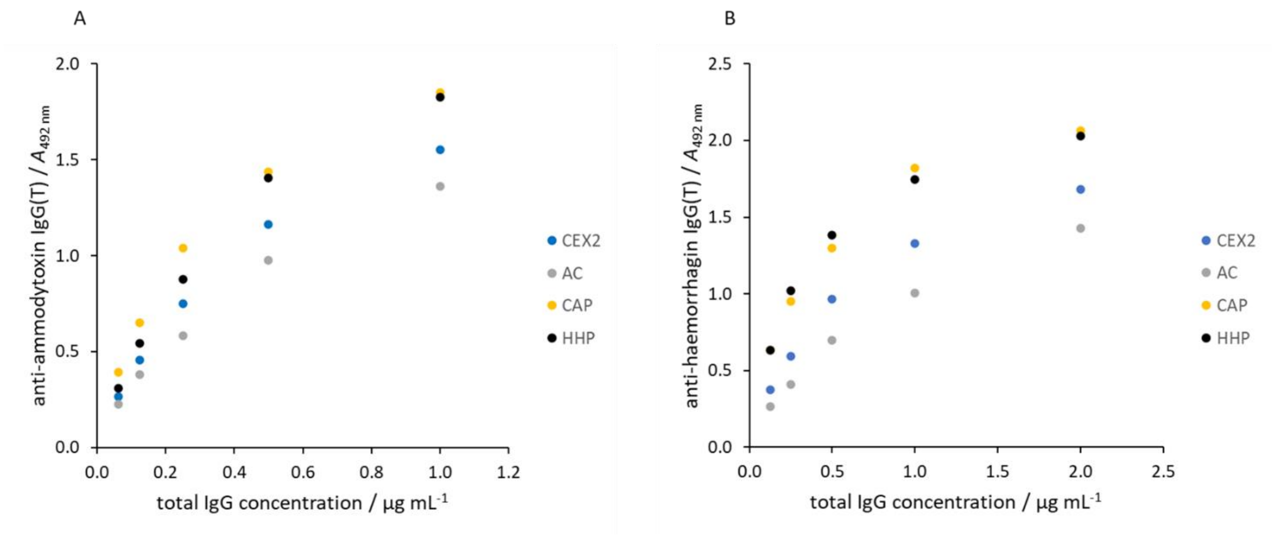

Figure S2. Ammodytoxin- and haemorrhagin-specific IgG(T) subclass determination in purified IgG fractions. Ammodytoxin (Atx) (**A**)- and haemorrhagin (H)-specific IgG(T) subclass (**B**) were determined in protein A affinity chromatography (AC), cation-exchange chromatography (CEX2) and caprylic acid precipitation (CAP) samples in comparison to hyperimmune horse plasma (HHP) determined by ELISA. Atx- or H-coated microtiter plates were incubated with IgG preparations and HHP prepared in equal total IgG concentration. Anti- IgG(T) conjugated with HRP was added to visualise Atx- and H- specific antibodies of only IgG(T) subclass.

Table S1. List of proteins identified by MS/MS analysis in the IgG preparations after ASP, AEX, CEX, CAP and AC purification processing.

Sample Lane	Gel Piece	[M+H]+ Peptide	Protein Name	Protein ID	Protein Score	Peptide Sequence
ASP1	1	1775.98	immunoglobulin gamma 5 heavy chain constant region ,partial	CAC86340.1	82	R.VPQVYVLAPHPDELAK.N
		1806.04	immunoglobulin gamma 5 heavy chain constant region ,partial	CAC86340.1	54	R.VVSVLPIQHQDWLSGK.E
			immunoglobulin gamma 4 heavy chain, partial	AAS18415.1		
			immunoglobulin gamma 2 heavy chain constant region, partial	CAC44761.1		
			immunoglobulin gamma 4 heavy chain constant region, partial	CAC44762.1		
	6	1992.98	histidine-rich glycoprotein	XP_023479252.1	67	K.DSPVLFDFFEDTELYR.Q
	7	2048.99	alpha-1B-glycoprotein	XP_023507318.1	105	R.VSFHLDALAPGDGGLYTCR.Y
		2854.40			105	R.REGDDQFLEVAEAPEDVVVTFPVHR.A
	8	2319.18	serum albumin precursor	NP_001075972.1	105	K.LPESERLPCSENHLALALNR.L
ASP2	13	2335.12	immunoglobulin alpha constant heavy chain, partial	AAP80145.1	62	R.LSGKPTHVNVSVVMAEADGTCY
		2642.18	immunoglobulin gamma 5 heavy chain constant region, partial	CAC86340.1	109	K.DFYPPEIDVEWQSNEHPEPEGK.Y
	14	1541.73	immunoglobulin gamma 4 heavy chain constant region, partial	CAC44762.1	63	K.DFYPTDIDIEWK.S
			immunoglobulin gamma	CAC44763.1		

			7 heavy chain constant region, partial			
		2642.20	immunoglobulin gamma 5 heavy chain constant region, partial	CAC86340.1	123	K.DFYPPEIDVEWQSNEHPEPEGK.Y
	15	1763.87	immunoglobulin gamma 4 heavy chain constant region, partial	CAC44762.1	98	K.SQTYICNVAHPASSTK.V
			immunoglobulin gamma 7 heavy chain constant region, partial	CAC44763.1		
			immunoglobulin gamma 6 heavy chain constant region, partial	CAC86341.1.1		
		2646.22	immunoglobulin gamma 5 heavy chain constant region, partial	CAC86340.1	80	K.DFYPPEIDVEWQSNEHPEPEGK.Y
	16	2048.99	alpha-1B-glycoprotein	XP_023507318.1	63	R.VSFHLDALAPGDGGLYTCR.Y
		2698.32			155	R.EGDDQFLEVAEAPEDVVVTFPVHR.A
		2747.46			105	R.THAAGDPSGPSAPVTIEELAAPPPPVLR.V
AEX1	19	1541.73	immunoglobulin gamma 4 heavy chain constant region, partial	CAC44762.1	84	K.DFYPTDIDIEWK.S
			immunoglobulin gamma 7 heavy chain constant region, partial	CAC44763.1		
		2143.01	immunoglobulin gamma 5 heavy chain constant region, partial	CAC86340.1	110	K.YSTTPAQLNSDGSYFLYSK.L
			immunoglobulin gamma 4 heavy chain constant	CAC44762.1		

			region, partial			
			immunoglobulin gamma 7 heavy chain constant region, partial	CAC44763.1		
	20	1994.83	serotransferrin precursor	NP_001075415.2	78	K.CACSNHEPYFGYSGAFK.C
		2686.32	transferrin, partial	AAF05482.1	79	K.HSTVLENLPQEADRDEYQLLCR.D
		2714.37			94	R.SVDGKEDLIWGLLNQAQEHFGTEK.S
	21	1097.50	serotransferrin precursor	NP_001075415.2	58	R.YYGYTGAFR.C
		2301.10	transferrin, partial	AAC78365.1	101	K.AVTEFESCNLAEAPNHAVVSR.K
	22	1178.56	histidine-rich glycoprotein	XP_023479252.1	63	R.HFGHPFHSGR.H
		1992.92			157	K.DSPVLFDFFEDTELYR.Q
	23	2698.33	alpha-1B-glycoprotein	XP_023507318.1	149	R.EGDDQFLEVAEAPEDVVVTFPVHR.A
	24	1932.05	serum albumin precursor	NP_001075972.1	60	K.SLHTLFGDKLCTVATLR.A
		2319.29			62	K.LPESERLPCSENHLALALNR.L
		2431.15			124	K.NCDLFEEVGEYDFQNALIVR.Y
	25	2291.08	beta-2-glycoprotein 1	XP_001499728.2	59	K.TSYVPGEEIVYSCQPGYVSR.G
AEX2	29	1763.82	immunoglobulin gamma 4 heavy chain constant region, partial	CAC44762.1	100	K.SQTYICNVAHPASSTK.V
			immunoglobulin gamma 7 heavy chain constant region, partial	CAC44763.1		
			immunoglobulin gamma 6 heavy chain constant region, partial	CAC86341.1		
		1794.02	immunoglobulin gamma 7 heavy chain constant region, partial	CAC44763.1	84	R.VVSILAIQHKDWLSGK.E
		1805.98	immunoglobulin gamma	CAC44762.1	72	R.VVSVLPIQHKDWLSGK.E

			immunoglobulin gamma 4 heavy chain constant region, partial			
			immunoglobulin gamma 2 heavy chain constant region, partial	CAC44761.1		
			immunoglobulin gamma 5 heavy chain constant region, partial	CAC86340.1		
CEX1	34	3049.59	predicted: alpha-2-macroglobulin-like	XP_008534363.1	83	R.EVLESLTEEAVKEDNSVHWTRPQKPK.A
	35	1654.89	predicted: alpha-2-macroglobulin-like	XP_008534363.1	88	R.AEHPFIVEEFVLPK.F
		1828.91			108	K.QLTFPLSSEPFQGSYK.V
		2518.08			160	K.VYDYYETDEFAIAEYNAPCGK.D
		3049.58			98	R.EVLESLTEEAVKEDNSVHWTRPQKPK.A
	38	2037.07	immunoglobulin gamma 1 heavy chain constant region, partial	CAC44624.1	63	R.SQEPQVYVLAPHPDELSK.S
		2920.40	immunoglobulin gamma 7 heavy chain, partial	AAS18414.1	64	K.ECGGCPTCPECLSVGPSVFIFPPKPK.D
	43	1914.03	hemopexin	XP_005612174.1	91	R.FNPVSGEVPPKYPLDVR.D
		2752.37			111	K.SGAAATWTELPWPHEKVDGALCVEK.S
	44	2319.15	serum albumin precursor	NP_001075972.1	76	K.LPESERLPCSENHLALALNR.L
		2431.09			178	K.NCDLFEEVGEYDFQNALIVR.Y
		2559.19			175	K.K NCDLFEEVGEYDFQNALIVR.Y
CEX2	48	1763.83	immunoglobulin gamma 4 heavy chain constant region, partial	CAC44762.1	82	K.SQTYICNVAHPASSTK.V
			immunoglobulin gamma 7 heavy chain constant region, partial	CAC44763.1		

			immunoglobulin gamma 6 heavy chain constant region, partial	CAC86341.1		
CAP	53	1804.61	immunoglobulin gamma 4 heavy chain constant region, partial	CAC44762.1	56	R.TISKPTGQPR.E
			immunoglobulin gamma 7 heavy chain constant region, partial	CAC44763.1		K.TISKPTGQPR.E
		1763.84	immunoglobulin gamma 4 heavy chain constant region, partial	CAC44762.1	85	K.SQTYICNVAHPASSTK.V
			immunoglobulin gamma 7 heavy chain constant region, partial	CAC44763.1		
			immunoglobulin gamma 6 heavy chain constant region, partial	CAC86341.1		
		2037.04	immunoglobulin gamma 1 heavy chain constant region, partial	CAC44624.1	61	R.SQEPQVYVLAPHPDELSK.S
	54	1114.61	alpha-1B-glycoprotein	XP_023507318.1	76	R.ALWSGAVTPGR.D
		1877.99			84	R.HWADLVLSYVGPQHAGK.Y
		1935.14			59	R.VQEASAQVLRPGVSLRPK.C
		2747.47			194	R.THAAGDPSGPSAPVTIEELAAPPPPVLR.V
		3035.55			107	K.DGVAQEPVQVGSPTIEHQFPLGAVTSDTR.G
	55	2431.15	serum albumin precursor	NP_001075972.1	89	K.NCDLFEEVGEYDFQNALIVR.Y
		2504.24	albumin, partial	AAG40944.1	106	K.LKPEPDAQCAAFQEDPDKFLGK.Y
		2504.24	serum albumin precursor	NP_001075972.1	105	K.LKPEPDAQCAAFQEDPDKFLGK.Y

AC	59	1378.75<br>1615.83<br>2188.96	immunoglobulin gamma 1 heavy chain constant region, partial	CAC44624.1	60<br>83<br>161	K.VNNQALPQPIER.T<br>R.IQHQDWLSGKEFK.C<br>K.YSTTQAQQDSDGSYFLYSK.L
		1775.97	immunoglobulin gamma 5 heavy chain constant region, partial	CAC86340.1	88	R.VPQVYVLAPHPDELAK.N
	60	1378.77<br>1615.85<br>2037.07<br>2189.00	immunoglobulin gamma 1 heavy chain constant region, partial	CAC44624.1	69<br>74<br>94<br>129	K.VNNQALPQPIER.T<br>R.IQHQDWLSGKEFK.C<br>R.SQEPQVYVLAPHPDELSK.S<br>K.YSTTQAQQDSDGSYFLYSK.L
	61	1378.76<br>1615.85<br>2037.06	immunoglobulin gamma 1 heavy chain constant region, partial	CAC44624.1	58<br>72<br>92	K.VNNQALPQPIER.T<br>R.IQHQDWLSGKEFK.C<br>R.SQEPQVYVLAPHPDELSK.S
	62	1541.72	immunoglobulin gamma 4 heavy chain constant region, partial	CAC44762.1	75	K.DFYPTDIDIEWK.S
			immunoglobulin gamma 7 heavy chain constant region, partial	CAC44763.1	75	K.DFYPTDIDIEWK.S
		1880.99	immunoglobulin gamma 7 heavy chain, partial	AAS18414.1	61	R.EPQVYVLAPHRDELSK.N
			immunoglobulin gamma 4 heavy chain, partial	AAS18415.1	61	R.EPQVYVLAPHRDELSK.N
		2037.03	immunoglobulin gamma 1 heavy chain constant region, partial	CAC44624.1	65	R.SQEPQVYVLAPHPDELSK.S

Table S2. Summary of quality-related properties of IgGs purified by ammonium sulphate precipitation (ASP), anion-exchange chromatography (AEX), cation-exchange chromatography (CEX), caprylic acid precipitation (CAP) and protein A affinity chromatography (AC).

Purification Procedure	Purity (%)	Retained Impurities	Aggregate Content (%)	Melting Temperature of Pure IgG (°C)	Melting Temperature of Pure IgG + Sorbitol (°C)	IgG Subclass Distribution Shift (Factor of Increase (↑) / Decrease (↓))	Specific Activity [*R** / γ(IgG)]
ASP1	83.1 ± 1.5	histidine-rich glycoprotein<br>alpha-1B-glycoprotein<br>albumin	4.5 ± 1.4	-	-	IgGa ↑ 1.06<br>IgGb ↑ 1.33<br>IgG(T) ↓ 1.25	-
ASP2	96.6 ± 1.2	alpha-1B-glycoprotein	1.4 ± 0.2	70.21	76.82	-	-
AEX1	76.7 ± 0.8	transferrin<br>histidine-rich glycoprotein<br>alpha-1B-glycoprotein<br>albumin<br>beta-2-glycoprotein 1	0.0 ± 0.0	-	-	IgGa ↑ 1.51<br>IgGb ↑ 1.54<br>IgG(T) ↓ 2.02	-
AEX2	98.0 ± 0.8	alpha-1B-glycoprotein	0.4 ± 0.1	70.38	76.51	-	-
CEX1	72.6 ± 2.2	alpha-2-macroglobulin-like<br>transferrin<br>hemopexin<br>albumin	5.4 ± 0.9	-	-	IgGa ↑ 1.42<br>IgGb ↑ 2.44<br>IgG(T) ↓ 2.65	-

CEX2	97.8 ± 0.8	-	1.2 ± 0.3	70.60	76.08	-	0.45
CAP	93.3 ± 1.4	alpha-1B-glycoprotein albumin	2.3 ± 0.3	69.69	74.98	IgGa ↓ 1.52<br>IgGb ↓ 1.17<br>IgG(T) ↓ 1.11	1.07
AC	92.1 ± 1.7	-	6.4 ± 1.4	68.54	73.4	IgGa ↑ 10.06<br>IgGb ↑ 2.55<br>IgG(T) ↓ 3.96	<0.41

* neutralisation potency expressing the number of LD_{50} doses that can be neutralised by 1 mL of pure IgG preparation.
